# A Case Report of a Child with Constipation Diagnosed with Acquired Myenteric Hypoganglionosis

**DOI:** 10.3390/reports8030108

**Published:** 2025-07-15

**Authors:** Niharika Singh, James Petrancosta, Elizabeth O’Daniel, Samuel Nurko, Kristen Calabro

**Affiliations:** 1 Department of Surgery, Stony Brook University Hospital, Stony Brook, NY 11794, USA; 2 Department of Surgery, Boston Children’s Hospital, Boston, MA 02115, USA

**Keywords:** acquired myenteric hypoganglionosis, pediatric surgery, constipation, case report

## Abstract

**Background and Clinical Significance:** Acquired myenteric hypoganglionosis is a rare dysmotility disorder that can present in childhood and adulthood, characterized by a reduced number of ganglion cells within Auerbach’s plexus. Due to the rarity of the pathology, few case reports of acquired myenteric hypoganglionosis in adolescents have been described. This case report explores the presentation, risk factors, and surgical complications associated with the ultimate diagnosis of myenteric hypoganglionosis. **Case Presentation:** We present a case of a 12-year-old male with a history of constipation and achalasia, presenting with constipation and abdominal distention, who underwent a colonoscopy, which was converted to an exploratory laparotomy with loop ileostomy creation due to persistent significant abdominal distention. This was complicated by colonic perforation, most likely secondary to stercoral colitis, requiring takeback to the operating room on postoperative day 11 for an exploratory laparotomy with bowel resection and mucous fistula creation. The patient was then referred to Boston Children’s Hospital for motility studies, which revealed poor colonic motility and plans to reassess motility in 1 year. **Conclusions**: Although rare, it is important to have high clinical suspicion for acquired myenteric hypoganglionosis in children, especially males, with severe constipation.

## 1. Introduction and Clinical Significance

Myenteric hypoganglionosis is a rare disease characterized by a reduction in the number of ganglion cells and diminished proliferation of glial cells within the myenteric (Auerbach) plexus [1]. This condition is considered a subset of isolated hypoganglionosis, which, as a whole, accounts for approximately 5% of all neuronal intestinal abnormalities [2]. Isolated hypoganglionosis is classified within the category of diseases known as the allied disorders of Hirschsprung’s disease, which each share clinical features with Hirschsprung’s disease, including persistent symptoms of severe intestinal obstruction, such as abdominal pain, vomiting, and distention. However, unlike Hirschsprung’s disease, these conditions exhibit some degree of ganglion cell presence.

Myenteric hypoganglionosis can present as either a congenital or an acquired condition. Congenital myenteric hypoganglionosis typically presents during the neonatal period and is thought to arise from inborn hypoplasia of the parasympathetic myenteric plexus [3]. In contrast, acquired myenteric hypoganglionosis tends to present at older ages (e.g., school-age [4] or adulthood [5]) and has been associated with a variety of etiologies, including viral infections and immune system dysfunction [6,7,8]. Both the congenital and acquired forms of myenteric hypoganglionosis can affect any segment of the gastrointestinal tract from mouth to anus, with a distribution that can be either patchy or contiguous [9,10]. This variability can pose a significant clinical challenge in identifying the precise location for bowel resection and ensuring complete excision.

Due to the clinical overlap with Hirschsprung’s disease and other Hirschsprung-related disorders, a definitive diagnosis of myenteric hypoganglionosis requires multiple full-thickness biopsies or extensive pathological examination of resected bowel segments [4,11]. The diagnosis is confirmed by the presence of a reduced number of ganglion cells within Auerbach’s plexus, in conjunction with a clinical presentation indicative of defective motility and intestinal dilation [5]. In the acquired form of myenteric hypoganglionosis, pathology is expected to reveal gliosis and a reduced quantity of ganglion cells within the myenteric plexus, with preservation of plexus size. This finding suggests that the myenteric plexus initially contained ganglion cells, which have since been destroyed [4,12]. In contrast, congenital myenteric hypoganglionosis is characterized by a diminished plexus with a low ganglion cell count, reflecting the incomplete development of the plexus. Given the rarity of documented cases, a definitive cutoff for the number of ganglia present required for diagnosis of hypoganglionosis has been difficult to establish [13]. However, advancements in immunostaining techniques using Hu C/D and CD56 have improved the accuracy of ganglion cell counting and have contributed to the recent development of novel diagnostic criteria [12,14]. No clear cutoff value to define myenteric hypoganglionosis exists due to small sample sizes and chronic distention of aganglionic segments, which alter density, but one source suggests that the loss of 2–3 ganglion cells per 1 mm of intestine is consistent with the diagnosis of myenteric hypoganglionosis [12]. Taguchi et al. demonstrated that patients who had hypoganglionosis had an average of 9.1 ± 5.4 ganglion cells/centimeter of intestine, which is about 24% that of normal bowel [1].

Treatment of myenteric hypoganglionosis often involves surgical resection of the affected bowel segments. The acquired subset of myenteric hypoganglionosis generally exhibits more favorable outcomes following surgical treatment when compared to congenital myenteric hypoganglionosis [4]. Although stem-cell-based treatment options using enteric nervous system progenitor cells are currently being explored, their application has been limited by the heterogeneous distribution of dysfunctional segments in acquired myenteric hypoganglionosis [15]. Emerging treatments utilizing enteric nervous system progenitor cells [16], mesenchymal stem cells [17], and combination mesenchymal stem cell/fecal microbiota transplantation [18] are currently being developed in murine models. However, these therapies are still in the early stages of development and require further investigation to evaluate their efficacy.

We present one of the few cases in the literature regarding myenteric hypoganglionosis. To our knowledge, this is the only case report that describes, in detail, the surgical complication of colonic perforation after initial diversion for myenteric hypoganglionosis. A 12-year-old male presented with chronic constipation who underwent unsuccessful colonoscopy, manual fecal disimpaction under anesthesia, and endoscopic decompression, ultimately requiring exploratory laparotomy with enterostomy creation, with further hospital course complicated by ascending colonic perforation and subsequent takeback to the operating room. Pathology of a resected segment of terminal ileum and colon was consistent with myenteric hypoganglionosis.

## 2. Case Presentation

The patient was a 12-year-old Caucasian male with a history of achalasia status post laparoscopic Heller myotomy with Dor fundoplication two years prior who presented as a transfer from an outside hospital for constipation and abdominal distention. The patient suffered from longstanding constipation at baseline, taking lansoprazole, MiraLAX, and Dulcolax, but usually had 1–2 bowel movements a week. At the time he was transferred to our institution, the patient had not had a bowel movement for 1 month. However, the patient had been passing flatus. A colonoscopy at the outside hospital was technically unsuccessful due to significant stool burden. The patient was born in the U.S. and lived in New York, with no recent travel outside the U.S. His family history was significant for a mother with multiple sclerosis and a paternal cousin with achalasia. The patient had no allergies and was up to date on vaccinations. He was born full-term without complications in the neonatal period, including normal meconium passage at birth. Esophageal manometry two years ago was consistent with type 1 achalasia, and an endoscopy at the time showed congested mucosa of the esophagus, which was biopsied and negative for any microorganisms. On presentation, labs were within normal limits. Physical exam revealed a largely distended, taught abdomen, that was not peritoneal. On digital rectal exam, there was no stool in the rectal vault.

**Figure 1:**
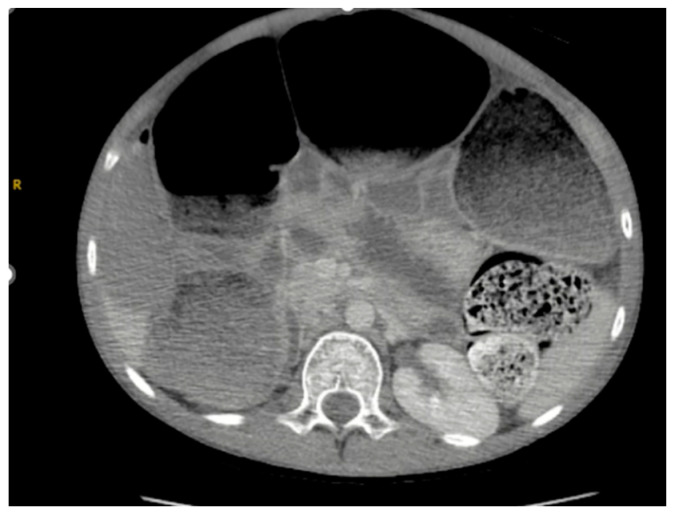
KUB on patient presentation, revealing a dilated transverse colon with significant stool burden.

**Figure 2:**
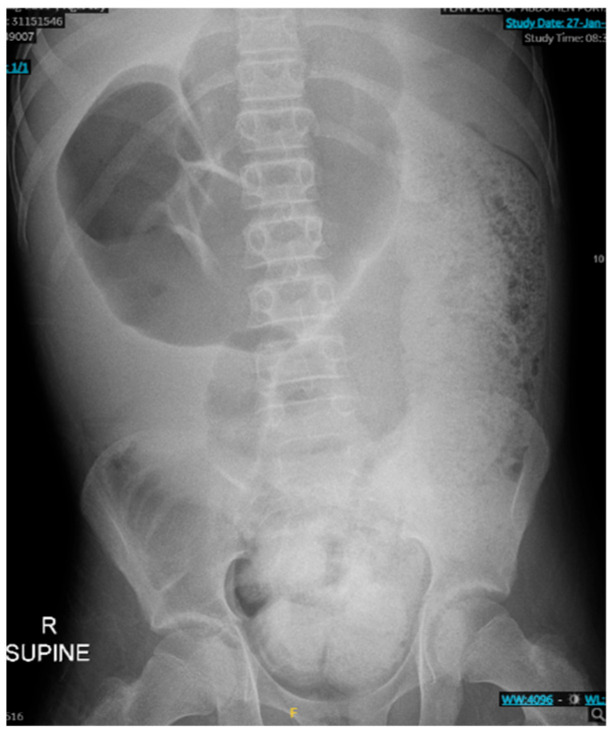
CT scan on patient presentation, revealing a colon dilated to 6.34 cm in diameter, with significant stool burden.

**Figure 3:**
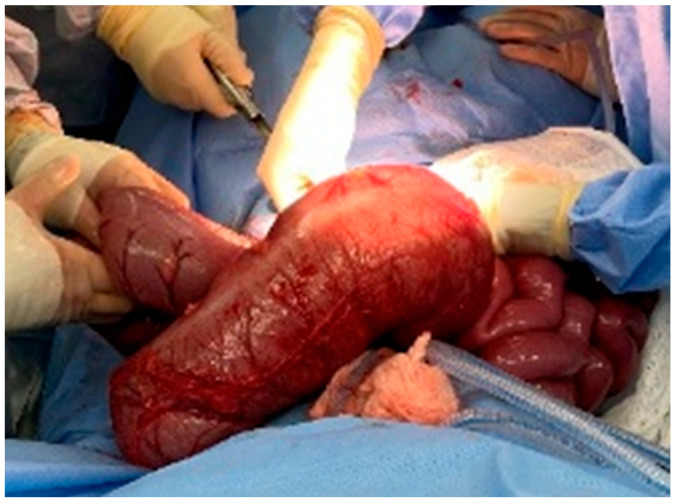
Dilated transverse colon in the initial exploratory laparotomy.

As seen in Figure 1 and Figure 2, the patient had a significantly dilated colon, measuring 6.34 cm in diameter. The patient was taken to the operating room for planned fecal disimpaction under anesthesia. The stool was too proximal to be accessed. At this point, neostigmine was administered without success. Gastroenterology was consulted intraoperatively to perform an endoscopic decompression. The colonoscopy was advanced 80 cm and revealed left-sided, thick, clay-like stool. The scope was passed beyond the stool burden, which allowed for the passage of a significant amount of air, but the abdomen continued to remain distended and tense. Due to the patient’s significant distention and concern for underlying dysmotility, an exploratory laparotomy was performed. This revealed a significantly dilated small and large bowel (Figure 3). An enterotomy was created in the distal ileum, and the stool was milked proximally through the enterotomy. Then, a loop ileostomy was created. The distal ileum was sent to pathology and revealed intramucosal ganglion cells but was otherwise inconclusive.

On postoperative day 2, neostigmine was administered again, and the patient had a large bowel movement from the rectum and the ileostomy was productive. On postoperative day 7, the patient became febrile with a leukocytosis of 19, so a CT abdomen pelvis was obtained with showed ascites. By postoperative day 10, the patient became tachycardic with a heart rate in the 120 s and worsening leukocytosis to 27, so interventional radiology (IR) was consulted and a 10 Fr draining catheter of the ascites was placed, the output of which was serous appearing. The day after the drainage procedure, the patient developed worsening abdominal distention and pain, tachycardia, and tachypnea, and the drain was acutely noted to have feculent output.

**Figure 4:**
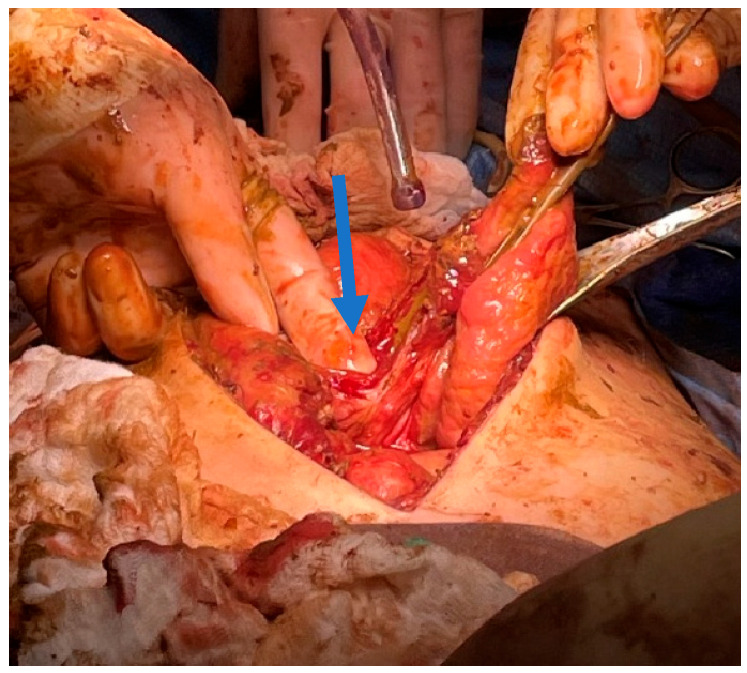
Arrow indicates perforation of the ascending colon (with surgeon’s finger inside) and stool in the peritoneum upon takeback.

At this point, the patient was taken to the operating room for an exploratory laparotomy which showed a large amount of stool in the peritoneum and gross perforation the ascending colon (Figure 4). At this point, part of the terminal ileum, cecum, and part of the ascending colon were resected without takedown of the initial ileostomy, and a mucous fistula was created from the ascending colon. Intraoperatively, the ascending colonic perforation appeared to be due to an inspissated stool. Postoperatively, the patient had an abscess drained by IR day 8 after the takeback operation. The patient’s hospital course is summarized in Table 1.

Pathology of the resected ileum, cecum, and ascending colon showed myenteric hypoganglionosis. Infectious disease was consulted and had low suspicion for Chagas disease due to the lack of epidemiologic risk factors and the results of the pathology. The patient was referred to Boston Children’s Hospital for follow up regarding motility studies. Approximately 6 months postop, he returned for upper endoscopy, antroduodenal, and colonic motility testing. Antroduodenal manometry revealed rare antral contractions and 9–11 enteral contractions per minute, consistent with antral hypomotility and normal small intestinal motility. Colonic manometry only revealed few non-propagating contractions, with no response to provocation, which was consistent with total colonic neuropathy. Anorectal manometry was significant for normal resting pressure at the internal anal sphincter; however, relaxation was difficult to evaluate on multiple maneuvers.

One year postop, he returned for routine follow up and has been doing well overall. He is without difficulty swallowing and is stooling regularly through the end ileostomy. He is planned for repeat manometry 1 year from initial testing.

## 3. Discussion

In this case report, we describe a 12-year-old male with a history of achalasia and a history of constipation presenting as a transfer for severe constipation refractory to conservative measures, taken to the operating room for an exploratory laparotomy with loop ileostomy creation. He required takeback to the operating room on postoperative day 11 for mid-ascending colonic perforation, most likely secondary to stercoral colitis, requiring exploratory laparotomy with bowel resection and mucous fistula creation, found to have myenteric hypoganglionosis on pathology.

The etiology of myenteric hypoganglionosis is not well understood, but may be due to ischemia, viral infections, multiple sclerosis, amyloidosis, or autoimmune conditions [6,7,8]. Although this patient did not have any personal history of autoimmune conditions or viral infections, his history was notable for a mother with multiple sclerosis. Additionally, a personal history of esophageal achalasia for patients later diagnosed with acquired hypoganglionosis has been described in prior case reports [1,19]. The pathology has a male predominance with a male-to-female ratio of about 3:1 [1,2]. The median age of diagnosis of myenteric hypoganglionosis described by Dingemann and Puri is younger—at about 5 years of age—than this case presentation [2].

The patient’s clinical presentation is consistent with presentations described in the literature. Taguchi et al. described four cases of acquired hypoganglionosis, all of whom had constipation that started at 5 to 10 years of age, with acute symptoms worsening after age 10 [1]. The location affected by hypoganglionosis ranges from total intestinal, total colonic, left hemicolonic, and rectosigmoid [2], with this patient’s motility results being most consistent with total colonic disease. Due to the lack of long term follow up studies, the prognosis of acquired hypoganglionosis is unclear but generally considered favorable. While some studies report the resolution of symptoms with the resection of the affected bowel, others report the need for additional operations due to the possible progression of disease versus the incomplete resection of the affected bowel at the index operation [1,4].

Treatment for acquired myenteric hypoganglionosis is the resection of the affected bowel, but the literature varies on the surgical approach—sources describe pull-through procedures, ileostomy or colostomy creation, and sphincter myectomy, all with favorable outcomes [1,2]. In a study describing adults with hypoganglionosis, most underwent total colectomy with ileorectal anastomosis, and few underwent enterostomy creation, but there was no recurrence of constipation with either treatment at 56 months post operatively [8]. We did not opt for the complete resection of a dilated colon at the index operation to allow for later manometric testing for the preservation of as much bowel as possible. The patient’s manometry testing is consistent with total colonic neuropathy, so ileocolonic anastomosis is not possible. Further anorectal manometry studies are necessary to completely evaluate internal sphincter relaxation. The patient and his family are satisfied with his ileostomy and do not want to pursue reversal at this time.

The limitations of this study include that it is a single case with lack of long-term follow up. However, this case highlights the presentation, risk factors, surgical options, and post operative management of the rare pathology myenteric hypoganglionosis.

## 4. Conclusions

Acquired myenteric hypoganglionosis is a rare dysmotility disorder that is important to consider in patients with persistent constipation. Risk factors may include ischemia, viral infections, and autoimmune disorders. It is important to have high clinical suspicion for this disorder in children, especially males, with worsening longstanding constipation and a personal history of other dysmotility disorders such as esophageal achalasia. Treatment includes resection of the affected bowel, which generally has positive outcomes. Outpatient manometric testing is helpful in evaluating the remaining bowel for consideration of performing an anastomosis in the future. The use of multidisciplinary care involving surgery, gastroenterology, infectious disease, and pediatrics is recommended in the management of this complex and rare pathology.

## Figures and Tables

**Table 1:** Summary of patient’s hospital course.

Hospital Day 0	Patient presents as a transfer; colonoscopy at outside hospital was unsuccessful
Hospital Day 1	Fecal disimpaction unsuccessful; colonoscopy performed but abdomen continued to be distended, so patient was taken for exploratory laparotomy and loop ileostomy creation
Hospital Day 8	Patient febrile with worsening leukocytosis, so IR places drain for ascites
Hospital Day 9	Feculent output noted from IR drain, so patient taken for exploratory laparotomy; ascending colon resection; mucous fistula creation
Hospital Day 17	IR performs abscess drainage
After Discharge	Patient underwent enteric manometry, which showed total colonic neuropathy

## Data Availability

The original contributions presented in this study are included in the article. Further inquiries can be directed to the corresponding author.
